# Effect of person-centred care on antipsychotic drug use in nursing homes (EPCentCare): study protocol for a cluster-randomised controlled trial

**DOI:** 10.1186/s13012-015-0268-3

**Published:** 2015-06-04

**Authors:** Christin Richter, Almuth Berg, Steffen Fleischer, Sascha Köpke, Katrin Balzer, Eva-Maria Fick, Andreas Sönnichsen, Susanne Löscher, Horst Christian Vollmar, Burkhard Haastert, Andrea Icks, Charalabos-Markos Dintsios, Eva Mann, Ursula Wolf, Gabriele Meyer

**Affiliations:** Institute of Health and Nursing Science, Medical Faculty, Martin Luther University Halle-Wittenberg, Halle (Saale), Germany; Institute of Social Medicine and Epidemiology, University of Lübeck, Lübeck, Germany; Institute of General Practice and Family Medicine, Faculty of Health, Witten/Herdecke University, Witten, Germany; Institute of General Practice, Medical Faculty, Heinrich-Heine-University Düsseldorf, Düsseldorf, Germany; mediStatistica, Neuenrade, Germany; Department of Public Health, Medical Faculty, Heinrich-Heine-University Düsseldorf, Düsseldorf, Germany; Institute of General Practice, Family Medicine and Preventive Medicine, Paracelsus Medical University, Salzburg, Austria; University Hospital of the Martin Luther University Halle-Wittenberg, Halle (Saale), Germany

**Keywords:** Implementation research, Nursing homes, Dementia, Antipsychotic agents, Person-centred care, Behavioural and psychological symptoms of dementia

## Abstract

**Background:**

The majority of nursing home residents with dementia experience behavioural and psychological symptoms like apathy, agitation, and anxiety. According to analyses of prescription prevalence in Germany, antipsychotic drugs are regularly prescribed as first-line treatment of neuropsychiatric symptoms in persons with dementia, although guidelines clearly prioritise non-pharmacological interventions. Frequently, antipsychotic drugs are prescribed for inappropriate reasons and for too long without regular reviewing. The use of antipsychotics is associated with adverse events like increased risk of falling, stroke, and mortality. The aim of the study is to investigate whether a person-centred care approach, successfully evaluated in nursing homes in the United Kingdom, can be implemented in German nursing homes and, in comparison with a control group, can result in a clinically relevant reduction of the proportion of residents with antipsychotic prescriptions.

**Methods/design:**

The study is a cluster-randomised controlled trial comparing an intervention group (two-day initial training on person-centred care and ongoing training and support programme) with a control group. Both study groups will receive, as optimised usual care, a medication review by an experienced psychiatrist/geriatrician providing feedback to the prescribing physician. Overall, 36 nursing homes in East, North, and West Germany will be randomised. The primary outcome is the proportion of residents receiving at least one antipsychotic prescription (long-term medication) after 12 months of follow-up. Secondary outcomes are residents’ quality of life, agitated behaviour, as well as safety parameters like falls and fall-related medical attention. A health economic evaluation and a process evaluation will be performed alongside the study.

**Discussion:**

To improve care, a reduction of the current high prescription rate of antipsychotics in nursing homes by the intervention programme is expected.

**Trial registration:**

ClinicalTrials.gov: NCT02295462

**Electronic supplementary material:**

The online version of this article (doi:10.1186/s13012-015-0268-3) contains supplementary material, which is available to authorized users.

## Background

Prescription rates of antipsychotic drugs for residents in German nursing homes are considerably high [1] and have not changed for years [2–5]. Recent data show that antipsychotics are prescribed for almost 30 % of German nursing home residents [1]. Studies indicate that up to two thirds of antipsychotics for residents are prescribed for inappropriate reasons [6]. Furthermore, antipsychotics are often prescribed for too long without carrying out a regular review [7]. Both long-term and short-term use of antipsychotics increase mortality compared to non-users [8–10], with cardiac death and infections being the main causes. All antipsychotics are associated with an increased risk of stroke, and the risk might even be higher in patients/residents receiving atypical antipsychotics compared to those receiving conventional antipsychotics [11]. Adverse effects also include sedation, dizziness and increased risk of falling and fall-related injuries, tremor and rigidity [12–16], worsening of cognitive function [17], and other effects which are most likely to result in reduced quality of life (QoL) and well-being as well as withdrawal from social life and participation.

Antipsychotics are mostly prescribed to control behavioural and psychological symptoms of dementia (BPSD) [1]. BPSD are a meaningful clinical target for interventions since restlessness, agitation, aggression, delusions, apathy, disinhibition, and hallucinations are highly prevalent among residents with dementia [18] and pose a major challenge for nursing home staff, frequently leading to distress in patients and carers [19]. The majority of residents with dementia experience BPSD at some point of the dementia trajectory, even in early stages of dementia [20, 21]. These symptoms are highly consistent over the course of five years with a tendency to increase over time [22]. International and national clinical practice guidelines recommend that psychological and environmental approaches should be the first treatment option of BPSD [23, 24], since non-pharmacological interventions individually tailored to the person with dementia are effective [25]. To meet individual needs of residents and improve their QoL, the person-centred care (PCC) approach seems to be appropriate. One highly recognised concept has been introduced by Kitwood [26, 27]. The components of this concept are to regard personhood in people with dementia as increasingly concealed rather than lost, to acknowledge the personhood of each individual in all aspects of care, to personalise care and environment, to offer shared decision-making, to interpret behaviour from the viewpoint of the person with dementia, and to prioritise the relationship as much as the care tasks [28]. Personalised activities were effective at reducing agitation in care home residents [29, 30]. Drugs should only be used as a last resort and should be discontinued immediately after symptoms vanish or improve over a period of 3 months [31, 32]. Thus, nursing homes are urgently requested to strive for a decrease of antipsychotics prescribed for BPSD in favour of non-pharmacological treatment [1].

Systematic reviews [33, 34] indicate that training and support for care home staff reduce prescription of antipsychotics in residents with dementia and can be viable alternatives for managing BPSD. The most recent and most rigorous trial [35], which is the basis for the present study, demonstrates the strongest effect without adverse events. In addition to a PCC approach, a review of drugs was conducted by an old-age psychiatrist taking part in this study [35]. After 12 months, the proportion of residents receiving antipsychotics in the intervention homes (23.0 %) was significantly lower than in the control homes (42.1 %) with an average reduction in antipsychotic use of 19.1 % (95 % confidence interval 0.5 to 37.7 %) [35].

Summarising, to evaluate the intervention programme by Fossey et al. [35] seems to be the most promising approach, although the programme requires a thorough adaptation to the German long-term care and health system.

## Trial objective

The aim of our study is to investigate whether a PCC approach, successfully evaluated in nursing homes in the United Kingdom [35], can be implemented in German nursing homes and, in comparison with a control group, can result in a clinically relevant reduction of the proportion of residents with antipsychotic prescriptions. Our *primary outcome* is the proportion of residents with at least one antipsychotic prescription (long-term medication) after 12 months of follow-up.

A further aim is to investigate whether QoL will improve or at least be unchanged as shown by Fossey et al. [35]. In accordance with the results of the primary efficacy study and a Cochrane review [33], we assume that the intervention is not only effective but also does not harm the residents, i.e. dementia-related BPSD will not worsen in the intervention group, and prescription rates of other psychotropic medication as well as the application of physical restraints will not increase. Therefore, the following *secondary outcomes* will be evaluated (at 12 months):

- Presence of agitated behaviour,
- Residents’ QoL,
- Median daily dose of antipsychotics in chlorpromazine equivalents,
- Proportion of residents with dementia with antipsychotic prescription,
- Proportion of residents without dementia with antipsychotic prescription,
- Prescription prevalence of other psychotropic drugs (antidepressants, anxiolytic and hypnotic drugs, acetyl-cholinesterase inhibitors),
- Safety parameters are number of falls, fall-related fractures and sutures, fall-related medical attention as well as the proportion of residents with at least one physical restraint (bilateral bedrails, belts, fixed tables, and other measures limiting free body movement), and the number and types of physical restraints.

Additionally, a health economic evaluation will be conducted alongside the study. A process evaluation will also be performed, aiming to comprehensively analyse the underlying processes as well as the barriers and facilitators of the implementation of this multicomponent intervention, giving special focus to regional differences. The programme will be implemented in nursing homes in East, North, and West Germany in order to evaluate its supra-regional generalisability.

## Methods/design

### Study design

**Fig. 1:**
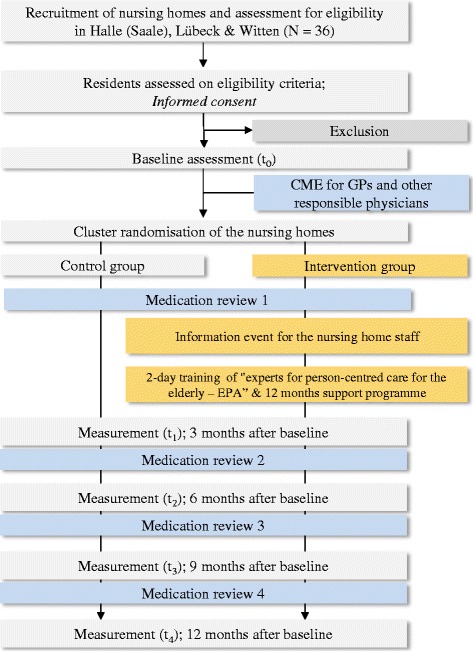
Flow chart of EPCentCare

The study is a multicentre, cluster-randomised controlled, pragmatic trial using parallel groups with a 1:1 randomisation (on cluster level) and 12 months of follow-up. Figure 1 displays the study flow.

The optimised usual care for both study groups will be a medication review by an experienced geriatrician and/or psychiatrist with feedback to the prescribing physician. All the general practitioners (GPs), neurologists, and psychiatrists for the study participants will be offered 2 h of continuing medical education on the subject.

For the nursing homes in the intervention group, an educational intervention on PCC and a continuous supervision programme will be conducted. The control group will receive no further intervention other than the optimised usual care.

### Participants and recruitment

#### Inclusion and exclusion criteria

The study will be performed in 36 nursing homes in the cities/catchment areas of Halle (Saale), Lübeck, and Witten. Individual nursing homes or units working independently within a large nursing home will be defined as a cluster.

Inclusion criteria will be nursing homes with at least 50 residents; each participating institution must have sufficient resources (staff, time) to conduct the study, and no special “dementia projects” or other activities potentially influencing the intervention will be allowed.

All residents within a cluster will be eligible to participate in the study. Exclusion criteria at the individual level will be:

- Temporary stay in respite care and/or
- Primary diagnosis of schizophrenia, or bipolar disorder.

#### Recruitment of study centres and study participants

In each study region, nursing homes will be randomly selected from the respective nursing home register. These facilities will be invited to take part in the study either via postal mail or telephone. The study will be presented to the directors of the nursing homes, who will decide whether their home will participate. A resident representative or, if not available, a residents’ advocate will be involved in all activities and decisions.

All eligible residents or their proxies or legal guardians will receive oral and written information about the aims and purpose of the study as well as all the information required according to good clinical practice (GCP) rules to create informed consent. Questions and additional information about the study will be available from the project staff of the respective study centre, either in person or by phone. Consent may be withdrawn for any reason at any time and without notice, without incurring any disadvantages for the residents. All residents who move into the nursing home during the study period will be eligible to participate too and will be asked for consent.

A total of four nursing homes (two facilities each in the intervention group and in the control group) will take part in a controlled pilot study at the study centre in Halle (Saale). The study will be implemented respectively in recruiting phases of four nursing homes per study site with a time shift of 2 months. The data collection will end in the last quarter of 2016.

### The EPCentCare intervention

The study intervention will consist of four components, depending on group assignment (see Table 1).

**Table 1:** Components of the study intervention

	Optimised usual care		Person-centred care (PCC) approach	
	Continuing medical education [23, 36]^a^	Medication review every 3 months [23, 35, 36, 39]^a^	Information for nursing home staff	Training and support of “experts for PCC for the elderly” [25–27, 29, 35, 40–43]^a^
Control group	x	x		
Intervention group	x	x	x	x

^a^Numbers in brackets indicate references concerning the theoretical and/or empirical background of study components

#### Continuing medical education provided for prescribing physicians (intervention and control group)

In Germany, residents of nursing homes have a free choice of medical practitioner. Therefore, the continuing medical education (CME) will address all physicians who are involved in the care of the participating residents of both study groups. The GPs, neurologists, psychiatrists, and geriatricians will take part in a 2-h workshop.

Topics of the training will be diagnostics and care for people with dementia as well as non-pharmacological and pharmacological treatment options for people with BPSD. The education content will be prepared in accordance with German national evidence-based clinical practice guidelines [23, 36]. Parts of the educational material have been used successfully in former studies [37, 38]. The focus will be on psychotropic medications and antipsychotics (e.g. guideline-based recommendations for use, adverse effects/risks, and contraindications). Furthermore, we will present and discuss options for optimising treatment and care for people with BPSD in nursing homes using the example of EPCentCare. All participants will receive written information about the central aspects of the training.

Physicians who do not attend the workshop, as well as new physicians joining the project during the study period, will receive written information on the workshop topics. The workshop will be offered locally at the participating universities.

#### Medication review (intervention and control group)

Residents of both study groups with an ongoing antipsychotic prescription will receive a medication review by a consultant psychiatrist or geriatrician. The medication review will be based on residents’ case files and communicated as a written report including specific recommendations to the prescribing physicians.

The medication reviews will be carried out at baseline and after 3, 6, and 9 months according to national evidence-based clinical practice guidelines [23, 36], the PRISCUS-list [39] and on the basis of product information documents. The respective relevant data for the psychiatrist/geriatrician will be pseudonymised. The study coordination centre will ensure forwarding of the reviews to the prescribing physicians.

#### Information event for the nursing home staff (only intervention group)

All the staff in intervention group homes will receive relevant information about the study in a 60-min presentation. They will get particular information on the topics “antipsychotics for people with BPSD” and “PCC and non-pharmacological interventions”.

#### Training and support of “experts for person-centred care for the elderly—EPA” (only intervention group)

Based on Fossey et al. [35, 40], selected staff from the nursing homes in the intervention group will be trained and instructed to work as “experts for person-centred care (PCC) for the elderly (EPA)”. The responsibilities of the EPA will be:

- Identifying organisational needs for change within the institution and opportunities for developing specific aspects of care
- Planning and supporting the implementation of PCC in the nursing facility.
- Initiating discussions with colleagues about PCC activities for specific residents.
- Discussion with colleagues about the medication review or contacting the prescribing physician.
- Advice and support of colleagues as facilitator and primary contact person for colleagues, relatives, and physicians.

The training programme for the EPA will include a 2-day initial workshop on PCC as well as continuous training and support over the 12-month study period.

The workshop will take place on two consecutive days at the respective study centre/university. An expert in PCC will present the following content:

- Introduction to EPCentCare.
- Antipsychotics in people with BPSD: list of medications, reasons for prescription, side effects, and guideline-based recommendations for use [23, 36, 41].
- PCC on resident level: according to Kitwood [26, 27], recognising individual residents’ needs and reasons for behaviour [40], dealing with challenging behaviour [25, 29, 40, 42, 43], and reflection of case examples.
- PCC on institutional level: assessing, supporting, and managing development of PCC in a nursing home [40, 41].
- Supervision/support programme [35].

The trained experts will be supported during the intervention period by a study nurse specialised in dementia and PCC (continuous in-house training). The supervision may be carried out individually and/or in groups in a minimum time frame of 3 to a maximum of 6 h per month and nursing home. As part of the supervision, the EPA will be supported in recognising residents’ needs [40] in implementing PCC plans, in promoting the participation of the residents in activities, and possibly in implementing environmental changes.

### Cluster-randomisation

The randomisation will be carried out on a cluster level. The randomisation list will be computer-generated by an independent external biometrician, who will be blinded to the identity of the participating organisations and residents. Nursing homes will be allocated to the intervention or control group using a balanced block randomisation, stratified by the three regions Halle (Saale), Lübeck, and Witten as well as the time point of the recruiting phase. Because of the low number of homes per stratum (*n* = 4), no further blocks within the strata are planned. This randomisation list will contain pseudonymous identification numbers for nursing homes and allocation of group A or B.

Parallel to this, responsible coordinators of the three study centres will independently determine and document the allocation (intervention group or control group) for group A and group B at study begin, without informing the external biometrician.

After completion of the baseline measurement of the corresponding recruiting phase, an independent person from the study centre in Lübeck will electronically (password-protected, encoded https portal) assign the randomisation list and nursing home data for concealed allocation [44] and will inform the nursing homes in written form. After confirmation of group allocation by the nursing homes, the assigning person will inform the coordinator of the respective study centre about the allocation results.

### Blinding

Due to the nature of the intervention, blinding of the nursing home staff and the field researchers will not be possible. However, the biostatistician, who will not be involved in conducting the study, will perform a step-wise statistical analysis blinded towards the group allocation of residents and clusters: (1) A blinded review of data without the nursing home IDs and group allocation will be carried out; depending on data structure, necessary adaptations of statistical analysis procedure will be performed. (2) Nursing home IDs and group allocation (group A or group B) are added to the data; group specific analyses will be carried out. (3) A or B will be unblinded after completion of the final analysis.

The psychiatrists/geriatricians performing the medication review are also blinded towards group allocation. Additionally, research assistants assessing the primary endpoint will be blinded towards group allocation. Data entry will be carried out without knowledge about the group allocation of the clusters.

### Measures

#### Institutional level

The description of the participating nursing homes, data on general characteristics, staff characteristics, characteristics of living environment, and living and care concept according to Palm et al. [45] will be gathered from the nursing home director before randomisation. The proportion of eligible residents receiving an antipsychotic prescription will be recorded at baseline and after 12 months.

#### Resident level

Five measurement points are scheduled in the study: baseline assessment and measurements after 3, 6, 9, and 12 months (see Table 2).

**Table 2:** Measurements at resident level

Point of measurement		Characteristics	Measure
t_0_	Baseline^a^	Socio-demographic and clinical data	Routine data
		Quality of life	QoL-AD
		Cognition	DSS
		Agitated behaviour	CMAI
		Medication data	Routine data
		Falls and fall-related medical attention	Routine data
		Physical restraints	Routine data
t_1_ t_2_ t_3_	3, 6, and 9 months after baseline	Medication data	Routine data
		Falls and fall-related medical attention	Routine data
		Physical restraints	Routine data
		Clinical course (emergencies, hospital admissions, change in nursing care dependency, and physician contacts)	Routine data
t_4_	12 months after baseline	Quality of life	QoL-AD
		Cognition	DSS
		Agitated behaviour	CMAI
		Medication data	Routine data
		Falls and fall-related medical attention	Routine data
		Physical restraints	Routine data
		Clinical course (emergencies, hospital admissions, change in nursing care dependency, and physician contacts)	Routine data

^a^New participants after t_0_ will be assessed with the same characteristics and measures as for baseline at the subsequent measurement point

At baseline (t_0_), socio-demographic and clinical data will be assessed from all included residents and will be documented in a case report form (CRF).

Residents’ QoL will be measured at t_0_ and t_4_ using the German version [46] of a QoL instrument which is also suitable for residents with dementia. The validated instrument *Quality of life-Alzheimer’s disease—QoL-AD* [47, 48] comprises 13 items with 4-point Likert scales for self or proxy assessment (total score ranges from 13 to 52, higher scores mean higher QoL). Self-assessment will be performed by short interviews with the residents; proxy assessment will be conducted by nursing staff.

To determine a cut-off point for the QoL-AD self or proxy assessment tool, the validated *Dementia Screening Scale* (*DSS*) [49] will be used as a cognition test (internal consistency: Cronbach’s alpha = 0.94). For this purpose, nursing staff will have to evaluate different aspects of cognitive function regarding seven items with 3-point Likert scales in the domains “memory” and “orientation” over the last 4 weeks (total score ranges from 0 to 14; higher scores indicate more severe cognitive impairment). As the test will be used to decide if a QoL-AD self-assessment is feasible, we will use a cut-off point of >4 (specificity 88.8 %, sensitivity 84.2 %) [49].

Agitated behaviour as one aspect of BPSD will be assessed with a German version [50] of the *Cohen-Mansfield Agitation Inventory* (*CMAI*) [51–53] (measurement points t_0_ and t_4_). The CMAI is a caregiver rating instrument with good psychometric properties of the original version [52] and will be performed by interviewing the nursing staff. The scale consists of 25 items of agitated behaviours, each rated on a 7-point scale of frequency of occurrence over the last 2 weeks (total score ranges from 25 to 175, higher scores mean higher frequencies of manifestation).

To analyse the proportion of residents receiving an antipsychotic prescription, the median daily dose of antipsychotic drugs in chlorpromazine equivalents, and the prescription prevalence of other psychotropic drugs, the following data will be gathered: (1) prescriptions of antipsychotics (long-term and pro re nata (PRN) medication) from routine documentation at t_1_, t_2_, t_3_, and t_4_ retrospectively for the last 3 months: registered trade name or agent, dose, and prescription period; (2) current prescriptions of other psychotropic drugs (long-term and PRN medication) from routine documentation at the time point of t_1_, t_2_, t_3_, and t_4_: registered trade name or agent, and dose.

Additionally, safety and/or adverse outcome parameters like falls, fall-related fractures and sutures, fall-related medical attention, as well as the number and types of physical restraints [2] will be documented at t_1_, t_2_, t_3_, and t_4_ retrospectively for the last 3 months, using data extracted from routine documentation.

New participants during the study period will be assessed at the next measurement point after their nursing home admission: socio-demographic and clinical data, QoL and cognition (QoL-AD, DSS), and agitated behaviour (CMAI) at this point will be regarded as baseline data of the new participant. Medication data and safety parameters since nursing home admission will also be documented retrospectively.

#### Data for medication reviews

For residents with an antipsychotic prescription at the point of measurement (t_0_ to t_3_), we will assess all antipsychotic prescriptions during the last 3 months and provide the psychiatrist/geriatrician with this information for the medication review.

All data for medication reviews are summarised in Table 3.

**Table 3:** Summary of data for medication reviews

Category	Variable
Medication data^a^ Long-term medication (antipsychotic drugs)	• Registered trade name or agent
	• Dosage form and regimen
	• Daily dose
	• Prescription period
	• Prescribing physician
	• Indication
PRN medication (antipsychotic drugs)	• Registered trade name or agent
	• Dosage form
	• Number of received partial dose
	• Suggested maximum dose
	• Prescription period
	• Prescribing physician
	• Case of need
Further current long-term and PRN medication	• Registered trade name or agent
	• Dose
	• Regimen
Medical care^a^	• Psychopathological reports
	• Number of physician contacts (GP, neurologist, psychiatrist, and geriatrician) over the last three months and date of the last visit
	• Body weight: current and three months before
	• Intolerance of drugs
Neuropsychiatric symptoms^a^ (over the last 2 weeks)	• Delusion
	• Hallucination
	• Aggression
	• Verbal abnormality
	• Agitation
	• Depression
	• Anxiety
	• Apathy
	• Disinhibition
	• Irritability
	• Sleep disturbances
Potential adverse effects of antipsychotic drugs^a^ (over the last 2 weeks)	• Pain
	• Aberrant motor behaviour
	• Malposition
	• Repetitive behaviour
	• Tremor
	• Somnolence
	• Delirium
Socio-demographic and clinical data^b^	• Age
	• Gender
	• Body height
	• Relevant clinical diagnoses
	• Falls

^a^Source: routine documentation of residents

^b^Source: case report form

### Process evaluation

Since we will be investigating a complex intervention, a detailed process evaluation is essential [54, 55]. Process evaluation outcomes will be collected according to a framework for cluster-randomised trials of complex interventions suggested by Grant et al. [56]. Different methods will be used for data collection alongside the study: investigators’ documentation, questionnaires on staff knowledge and self-efficacy, structured interviews, and in-depth interviews with staff, residents, and their relatives. A detailed protocol is under preparation.

### Health economic evaluation

The objective of the economic evaluation is to estimate the cost-effectiveness of the intervention in terms of additional costs per additional resident not receiving an antipsychotic prescription. The economic evaluation will be performed from the perspective of the German social insurance (statutory health insurance and long-term care insurance). To this end, an incremental cost-effectiveness ratio (ICER) will be calculated, i.e. the ratio of the difference in costs between intervention and control group divided by the difference in the proportion of residents without a psychotropic medication gained in each group.

A detailed description of the health economic evaluation can be found in Additional file 1.

### Sample size calculation

The primary endpoint of the study is the proportion of residents with at least one antipsychotic prescription after 12 months (t_4_). The following sample size estimation was performed as described in Donner & Klar [57] for the cluster-adjusted *χ*^2^-test. Based on recent prevalence data, we expect a baseline prevalence of antipsychotic prescription of 30 % [2]. Due to the measures of optimised usual care in the control group and based on the study by Fossey et al. [35], we expect the prevalence in the control group to decline slightly to 26 % after 12 months. We expect a relative risk reduction of approximately 50 % regarding antipsychotic prescription in the intervention group.

Assuming a significance level of 5 % (α = 0.05), a two-sided test for proportions, and an intra-class correlation coefficient (ICCC) of 0.05, an average cluster size of 65 nursing home residents, 15 clusters are required per group to detect a 12 % difference in proportions from 26 % of the control group at 12 months with 90 % power (β = 0.10). Presuming that six nursing homes might drop out, 36 clusters with a total of 2340 residents will be needed.

We expect 25 % of residents with early study termination due to death or moving; the other 75 % of residents of the baseline population we expect to be still living in the nursing home and to be available for the 12-month follow-up assessment. As the outcome, the analyses will focus on the group of residents living in the nursing homes after 12 months. Assuming that new residents will all be willing to participate in the study, this will not affect the sample size.

### Statistical analysis

No interim analysis will be performed. Analysis will be based on the intention-to-treat principle. The analysis will be done at the resident level.

For the primary outcome measure, i.e. the proportion of residents with at least one antipsychotic prescription after 12 months, proportions will be compared between the intervention group and control group, using a two-sided cluster-adjusted *χ*^2^-test at a level of significance of α = 0.05 [57]. Corresponding cluster-adjusted 95 % confidence intervals will be calculated. Furthermore, to investigate the time course of the intervention effect, including all the residents observed after 3, 6, 9, or 12 months, drug prescriptions of antipsychotics per resident after 3, 6, 9, and 12 months will be analysed as a dependent variable in a generalised linear mixed (logistic) model with the intervention as a fixed effect, clusters as a random effect, and using covariance patterns to adjust for repeated measurement. This will be done as a secondary analysis.

For the secondary outcome measures, linear mixed models or generalised linear mixed methods will be used, adjusting for clusters by random effects. Subgroup analyses using linear mixed models will be performed for gender and cognitive status. The analysis of all secondary outcomes is interpreted exploratively and not confirmatively.

A detailed description of the statistical analysis can be found in Additional file 2.

### Data management

Study data will be recorded, pseudonymised in paper form in the nursing homes, and electronically stored afterwards. The personal data of the study participants (name, birthday, etc.) will be kept separately from the study data. All participating nursing homes will also receive a pseudonymous identification number. Key lists will be stored separately from the study data and will be deleted after final validation of the analytical data set and data freezing.

Data will be entered electronically at the study centre in Lübeck. The data matrix will be based on the codebook created prior to the study. The questionnaires will be read electronically by a scanner-based software (TeleForm®). During scanning and electronic recording, a plausibility check will be performed. Written requests will follow in the case of unclear data or lack of data. Unclearly placed check marks and text fields will be checked and corrected manually. To ensure data quality, important data (e.g. antipsychotic prescription) will be checked by hand. Changes and corrections to the database will be clearly documented. After data freezing, no further data changes will be possible. Data entry will be performed within 1 month after recording.

### Quality assurance

The study will be planned, implemented, and evaluated in accordance with the principles of good clinical practice (ICH-GCP) [58] and the Declaration of Helsinki [59].

A concordant study procedure in all three study centres will be ensured by a central external audit. Data monitoring will increase the credibility of the study and help to improve data collection and archiving procedures. It will be carried out according to a data-monitoring manual which follows GCP and which has been developed and applied in a former study [60].

### Dissemination policy

We plan to publish the study results in a peer-reviewed, Medline-listed journal. All results will be reported referring to this study protocol and to the CONSORT statement extended to cluster-randomised trials [61] as well as to CReDECI [62].

We will set up a German-language homepage for reporting the study results and accessing all materials, including the intervention programme, after the end of trial.

### Ethical and legal considerations

This project has received ethical approval from the ethics committee of the Medical Faculty, Martin Luther University Halle-Wittenberg, Germany (no. 2014–101), as well as from the ethics committees of the Universities of Lübeck (no. 14–239) and Witten/Herdecke (no. 133/2014). All ethics committees will be informed immediately about any protocol amendments and serious or unexpected adverse events as well as a premature end of the study.

## Discussion

Based on previous study results [2, 35], we do not expect any risks for the participating residents. The intervention programme has been tested successfully in the United Kingdom, and the discontinuation of antipsychotics showed no negative consequences [35]. Systematic reviews support only a short-term antipsychotic treatment, because of the significant adverse effects and a lack of evidence for prolonged prescription [17, 31]. We expect that the intervention programme will reduce the current high prescription rate of antipsychotics in nursing homes.

### Trial status

The pilot phase of the study has been started.

## Additional files

Additional file 1:
**Detailed description of the health economic evaluation.**
Additional file 2:
**Detailed description of the statistical analysis.**

## Abbreviations

BPSD: Behavioural and psychological symptoms of dementia
CMAI: Cohen-Mansfield Agitation Inventory
CME: Continuing medical education
CRF: Case report form
DSS: Dementia Screening Scale
EPA: Experts for person-centred care for the elderly
EPCentCare: Effect of person-centred care on antipsychotic drug use in nursing homes (acronym of the study)
GP: General practitioner
ICCC: Intra-class correlation coefficient
ICER: Incremental cost-effectiveness ratio
ICH-GCP: International Conference on Harmonisation—good clinical practice
PCC: Person-centred care
PRN: Pro re nata (“when required”)
QoL: Quality of life
QoL-AD: Quality of life in Alzheimer’s disease
