# Supplementary material for: Effect of person-centred care on antipsychotic drug use in nursing homes (EPCentCare): study protocol for a cluster-randomised controlled trial
Source: Implement Sci. 2015 Jun 4;10:82. doi: 10.1186/s13012-015-0268-3 (PMC4464611; doi:10.1186/s13012-015-0268-3)
Supplement: Additional file 1: — Detailed description of the health economic evaluation. [file 13012_2015_268_MOESM1_ESM.pdf]

Additional file 1:

Detailed description of the health economic evaluation

ClinicalTrials.gov Identifier: NCT02295462

The objective of the economic evaluation is to estimate the cost-effectiveness of the intervention in terms of additional costs per additional resident not receiving an antipsychotic prescription. The economic evaluation will be performed from the perspective of the German social insurance (statutory health insurance and long-term care insurance). To this end, an incremental cost-effectiveness ratio will be calculated, i.e. the ratio of the difference in costs between intervention and control group divided by the difference in the proportion of residents without a psychotropic medication gained in each group.

Effect parameters will be taken from the trial (see measures in main manuscript). Cost parameters will be collected alongside the study on intervention-related components as well as outcome-related components - based on cost protocols developed for earlier own studies [1-3]. For this, in addition to data of residents (e.g. medication, see measures), at points of measurement t_1 to t_4 we will retrospectively document the following aspects referring to the last three months: emergencies, hospital admissions, change in nursing care dependency, and physician contacts. All other cost parameters will be registered through the course of the study or during process evaluation. Healthcare resource use due to interventions and other reported healthcare utilization (consultations, hospital days, etc.) will be multiplied by unit costs/prices. Currently, there are no German guidelines for costing in economic evaluations containing standard unit costs. Hence, healthcare resource use will be valued by unit costs/prices obtained from published sources and official statistics for Germany (e.g. charges and rates from administrative databases, pharmacy retail prices). Resource use and costs directly associated with the intervention (e.g. staff time) will be derived from the study documentations. Costs explicitly associated with the conduct of the study, such as data collection or delivery of medication reviews, will not be taken into account.

For the health economic analysis, mean costs as well as cost differences between intervention and control group will be calculated. Adjustment for cluster correlation is planned, if possible. Sampling uncertainty (95 % confidence intervals) will be estimated using bootstrap procedure because cost data are usually non-normally distributed (skewed). Effects will be taken from the clinical analysis. Incremental cost-effectiveness ratio (ICER) will be estimated in terms of costs per additional resident without psychotropic medication. The non-parametric bootstrap

method will be employed to generate confidence intervals around the ICER estimates derived from the study sample [4, 5]. Uncertainty surrounding the ICER will also be presented on the cost-effectiveness plane [6, 7] and as the cost-effectiveness acceptability curve [8, 9]. Besides statistical uncertainty (sampling variation) with regard to costs and effects, every economic evaluation may contain some degree of data imprecision (e.g. resource costs/prices), which should be accounted for. To handle this type of uncertainty, sensitivity analysis will be employed. In the sensitivity analysis, (uncertain) parameter(s) of the base-case analysis will be varied to determine whether changes in these parameters influence the results. We will report both the revised point estimates and revised confidence intervals for costs, effectiveness, and cost- effectiveness that result from the sensitivity analyses.

References

1. Holt S, Schmiedl S, Thürmann PA: **Potentially inappropriate medications in the elderly: the PRISCUS list.** *Dtsch Arztebl Int* 2010, **107:**543-551.
2. Köpke S, Mühlhauser I, Gerlach A, Haut A, Haastert B, Möhler R, Meyer G: **Effect of a guideline-based multicomponent intervention on use of physical restraints in nursing homes: a randomized controlled trial.** *JAMA* 2012, **307:**2177-2184.
3. Meyer G, Köpke S, Haastert B, Mühlhauser I: **Comparison of a fall risk assessment tool with nurses' judgement alone: a cluster-randomised controlled trial.** *Age Ageing* 2009, **38:**417-423.
4. Briggs AH, Wonderling DE, Mooney CZ: **Pulling cost-effectiveness analysis up by its bootstraps: a non-parametric approach to confidence interval estimation.** *Health Econ* 1997, **6:**327-340.
5. Hunink MG, Bult JR, de Vries J, Weinstein MC: **Uncertainty in decision models analyzing cost-effectiveness: the joint distribution of incremental costs and effectiveness evaluated with a nonparametric bootstrap method.** *Med Decis Making* 1998, **18:**337-346.
6. Black WC: **The CE plane: a graphic representation of cost-effectiveness.** *Med Decis Making* 1990, **10:**212-214.
7. van Hout BA, Al MJ, Gordon GS, Rutten FF: **Costs, effects and C/E-ratios alongside a clinical trial.** *Health Econ* 1994, **3:**309-319.
8. Fenwick E, Claxton K, Sculpher M: **Representing uncertainty: the role of cost-effectiveness acceptability curves.** *Health Econ* 2001, **10:**779-787.
9. Fenwick E, O'Brien BJ, Briggs A: **Cost-effectiveness acceptability curves - facts, fallacies and frequently asked questions.** *Health Econ* 2004, **13:**405-415.
