# Supplementary material for: Effect of person-centred care on antipsychotic drug use in nursing homes (EPCentCare): study protocol for a cluster-randomised controlled trial
Source: Implement Sci. 2015 Jun 4;10:82. doi: 10.1186/s13012-015-0268-3 (PMC4464611; doi:10.1186/s13012-015-0268-3)
Supplement: Additional file 2: — Detailed description of the statistical analysis. [file 13012_2015_268_MOESM2_ESM.pdf]

Additional file 2:

Detailed description of the statistical analysis

ClinicalTrials.gov Identifier: NCT02295462

No interim analysis will be performed. Analysis will be based on the intention-to-treat principle. The analysis will be done at the resident level.

Baseline data will be analysed descriptively for the control group and the intervention group without statistical testing or cluster adjustment except for antipsychotics and general psychotropic prescriptions, which will be adjusted for cluster. The outcome analysis made after 12 months will consist of all residents of the nursing homes at this time, excluding residents who early terminated the study, but including new residents. Characteristics of the outcome analysis made will be described for both the control group and the intervention group.

For the primary outcome measure, i.e. the proportion of residents with at least one antipsychotic prescription after 12 months, proportions will be compared between the intervention group and control group, using a two-sided cluster-adjusted χ^2-test at a level of significance of $\alpha = 0.05$ [1]. Corresponding cluster-adjusted 95% confidence intervals will be calculated. Furthermore, to investigate the time course of the intervention effect, including all the residents observed after 3, 6, 9 or 12 months, drug prescriptions of antipsychotics per resident after 3, 6, 9 and 12 months will be analysed as a dependent variable in a generalised linear mixed (logistic) model with the intervention as a fixed effect, clusters as a random effect, and using covariance patterns to adjust for repeated measurement. This will be done as a secondary analysis.

For the secondary outcome measures (quality of life, dose of antipsychotic drugs in chlorpromazine equivalents, and prescription prevalence of other psychotropic drugs as well as safety outcome parameters), linear mixed models or generalised linear mixed methods will be used, adjusting for clusters by random effects. Subgroup analyses using linear mixed models will be performed for gender and cognitive status (presence vs. absence of dementia). The analysis of all secondary outcomes is interpreted exploratively and not confirmatively.

An additional analysis will be performed to investigate possible bias through early study termination. Times of early study termination will be compared between the intervention group and control group stratified by antipsychotic prescriptions at baseline by Kaplan-Meier curves and Cox regression. All residents at baseline assessment will be included in the

corresponding analysis population. The outcome event is early study termination because of death or moving, and the times from baseline assessment to early study termination will be evaluated. In the case of no early study terminations, the censored time is the time from baseline assessment to the last assessment, even if the resident was not documented at this time. Kaplan-Meier curves are stratified into four strata by intervention/control group and antipsychotic prescription at baseline yes/no. Cox regression will be performed using intervention/control, antipsychotics at baseline yes/no, and the interaction of both factors, with age and sex (possible confounders) as independent variables. Shared frailty is used for cluster adjustment in the Cox model.

All residents who move into the nursing home after the randomisation are eligible to participate and will be asked to do so. All eligible new residents will be recorded and compared with the proportion of residents who agreed to participate before randomisation in order to exclude a bias in inclusion of residents after randomisation. The subgroup of newly included residents (residents after t_0) will be analysed separately for exploratory purposes.

Few missing values are expected in the outcome measures. Residents with missing data are excluded from specific outcome analyses. The effect in this cluster randomised study will occur primarily on cluster level, so that a few missing data will not bias the results. No imputation analysis is planned. Furthermore, mixed models analyses on time courses will include all patients using all evaluable values in the outcomes. A Cox regression analysis will be performed to investigate possible bias by early termination.

References

1. Donner A, Klar N: *Design and Analysis of Cluster Randomization Trials in Health Research.* London: Arnold; 2000.
